# Post-Mortem Examination of the Kings of France, from Charles IX. To Louis XVIII

**Published:** 1830-01-01

**Authors:** 


### XLVII.

### Post Mortem Examinations of the Kings of France from Charles IX. to Louis XVIII. From Authentic Documents arranged by Dr. Henry Dupuy.*

There is a disposition inherent in the human mind to invest those removed from the common sphere of life, with attributes that appertain not to ordinary mortality. Who that reads the exploits of Alexander, can picture to himself the Macedonian demigod subject to those little corporeal annoyances that chafe the temper of Mr. Thompson or Mr. Smith—can imagine Hercules troubled with constipation of the bowels—or Julius Cæsar plagued with corns, albeit

*He* had a fever whilst he was in Spain!

But human nature is human nature still, however the grand, the moral, the intellectual *spiritus* may dazzle the eyes of the astonished world; and a hero and a costermonger suffer in no very unequal degree from those bodily inconveniences and ills to which flesh is heir. Sylla was destroyed by the lousy distemper — Napoleon le Grand, l'Invincible, as a foolish universe once thought its scourge, died of a *malignant* disease—and our own race of kings have notoriously suffered from the complaint which is usually the property of the squalid and the needy—scrofula. Some very curious and interesting documents have been published in France, respecting the examinations of the bodies of their kings, from Charles the IXth to the last Louis. Previously to the times of Charles the prejudices of the people and the opposition of the clergy restricted the examination of bodies to that of executed criminals. It was not to be supposed that the haughty sovereign of a feudal nation should descend to the level of felons, of those whom the ideas of the times would scarcely have ranked in the same class of beings as himself. It required some extraordinary event to establish the necessity for royal dissections, and such an event presented itself in the remarkable death of the ninth Charles. This Gaulish representative of the Neros and Domitians of the world, is execrable to all time by the massacre of the Hugonots at Paris, on St. Bartholomew's day, in 1572. When the hour for that dreadful outrage approached, being upbraided with indecision by the savage Catherine de Médicis his mother and the regent, he exclaimed, "well then let not one be left to reproach me with breach of faith"! He even fired with his own hand on the miserable wretches endeavouring to escape across the Seine. It was said that from this time to his death, which took place in May, 1574, he never enjoyed a tranquil hour, and various reports were bruited about respecting the mode of his decease. Many regarded the event as a punishment for his enormous crime, and asserted that he fell the victim to a *sweating of blood;* others on the contrary attributed it to the machinations and ambition of the Duke d'Alençon. In order to set at rest all rumours and dispel these suspicions, Catherine decided that the body of her dear son should be examined, and Charles was thus the first king of France, the first descendant of Charlemagne, whose body was profaned by the scalpels of his subjects. From that time to this, the examinations of their monarchs after death has become a matter of court-etiquette in the French dominions. Before we proceed to the account of the dissection of Charles, it will be interesting to recite a few particulars respecting his mortal illness.

Papyre Masson, a writer of those times, states that he fell ill in the month of October, 1573, whilst attending his brother, afterwards Henry III, on his departure for Poland. He was first attacked with pains in the chest, which were not at all understood by his medical attendants, and continued to in-

* Révue Médicale; Sept. 1829.

crease; he was worn down by an "erratic" fever, sometimes quartan, sometimes continued; and in spite of all that Mazille, his first physician, could do, the disease proved fatal. L' Etoile gives an interesting account of the last days of the suffering king. On the Friday, says he, preceding the Sunday when Charles died, about two o'clock in the afternoon, he called for Mazille, and, after complaining of the pain he endured, inquired if it was not possible for him, and the many other physicians whom he had in his kingdom to procure some alleviation of his miseries. Mazille replied, that all which depended on their art had been done, that the very day before the faculty had assembled for the purpose of giving relief, but that, to speak the truth, God was the only, and sovereign physician for such diseases to whom he could have recourse. "I believe," said the king, "that what you tell me is true, and that you know of nothing else. *Tirez-moi ma custode, que j'essaye à y reposer.*" It is reported by Guy Patin, and other writers, and with every appearance of probability, that poor Mazille narrowly escaped *hanging* by order of Catherine, for not having called a consultation sufficiently early. We cannot help thinking, that if some such plan were adopted now-a-days, it might save some patients at the expense of a doctor or two, and otherwise be attended with much service. The Latin account of the post mortem examination is curious.

"RAPPORT DU CORPS MORT DU FEU ROI CHARLES IX.

"'Anno domini miles, quinquent. septuag., quarto pridii kal. junii, hora a meride quarta, facta est dissectio corporis Caroli IX, regis Galliarum christ., assidentibus medicis hic subsignatis et chirurgis qui eam administrarunt, in qua accuratè hæc observata et deprehensa sunt.

"'1. Hepatis totum parenchyma arefactum, exangue, et extremis lobis ad simas partes vergentibus nigricans.

"'2. Folliculus fellis a bile vacuus, in sese considens, subater.

"'3. Lien nullo modo malè affectus.

"'4. In ventriculo nulla noxa, et stomachi cum pyloro integritas.

"'5. Intestinum colon flavum colorem contraxerat, cæteris benè habentibus.

"'6. Epiploum malè coloratum, supramodum extenuatum; parte aliquâ ruptum, et omnis pinguidinis expers.

"'7. Ren uterque nullo vitio obsessus, nullo similiter vesica, nullo uretres.

"'8. Cor flaccidum et velluti contabescens: omnis aquoso humore, qui pericardio contineri solet, absumpto.

"'9. Pulmo qui in partem sinistram thoracis incubebat, a costis illegitimis ad claviculas usque totus lateri adhærebat, ita firmiter et obstinatè, ut avelli potuerit sine dilaceratione, et discerptione cum putridine substantiæ, in qua sese prodidit vomica rupta, è qua colluvies purulenta, putrida et graveolens effluxit, cujus tanta fuit copia, ut in asperam arteriam redundarit, et præclusa respiratione præcipitis et repentini interitûs causam attulerit.

"'10. Alter pulmo sine adhæsu fuit, magnitudine tamen naturalem constitutionem, turgidus et distentus superans (ut et sinister superabat in substantia, insignem corruptelam præ se ferens) parte superiore putris, refertus et conspurcatus humore pituitoso, mucoso, spumoso, puri finitimo.

"'11. Cerebrum omni vitio carens."

"Medici qui præfuerunt,

"*Regii* Mazille. Vaterre. Alexis Gaudin. Vigor, Lefevre, Saint Pons.

"*Parisienses.* Piètre, Brigard, Lafilé et Duret.

"*Chirurgi regii qui administraverunt,* Paré, d'Ambroise, Portail, Eustache, Dioneau, Dubois, Lambert et Cointenel."

It appears to us from the foregoing account, that Charles IX. died of inflammation and suppuration of the left lung.

HENRI III.

This prince died from a wound inflicted by the knife of an assassin in the hypogastrium. He lived for about eighteen hours after the injury, and suffered from frequent fits of weakness, suffocation, fever, intolerable thirst, and the greatest agony. We learn by the notes of the dissection, that a portion of the ileon was pierced through and through by the knife, and that the mesentery was also wounded in two places, with incision of its vessels. The contents of the thorax, abdomen, and head, were otherwise healthy.

### Henri IV.

The Alfred of France, Henry the Great, was stabbed in his carriage on the 4th of May, 1610: he died almost immediately, after uttering a few words and discharging blood by the mouth.

On the left side of the chest, about the level of the second and third rib, was a wound capable of admitting the finger; it ran on the pectoral muscle towards the nipple for the length of four inches, but did not penetrate the chest. Below this was another wound between the fifth and sixth rib, about two fingers' breadth, penetrating the thorax, piercing one of the lobes of the left lung, and wounding the trunk of the pulmonary artery, a little below the left auricle. There was much blood extravasated in that side of the chest, and both lungs were filled with it.

### Louis XIII.

This document is written in Latin, and grâces de Dieu such Latin, by the *doyen* or dean of the Ancient Faculty of Medicine, which, from the dissection of the present King downwards, has been required to assist at the mournful ceremony.

A circumstance is related of this Louis on his death bed, which is worth transcribing. "When," says the historian, "his physician, at his earnest desire, numbered the fleeting minutes that remained, and pronounced that his life could not exceed two or three hours; he received the intelligence with resignation and even satisfaction; and looking fervently up to heaven, added, "Well! I consent with all my heart." Here is the account of the dissection.

"Postero autem die (id est 15 mensis maii 1643), hora sexta matutina defuncti regis cadaver apertum præsentibus serenissimo principe ac domino de *Nemours*, marescalco sive castrorum præfecto primario; domino de *Vitry*, domino de *Souvré*, primo cubiculario nobili sive inter nobiles, regi à cubiculis primario, medicis regis ac reginæ primariis, aliis quoque medicis et chirurgis, ex *utraque* familia chirurgorum Paris.... Atque in hoc regis cadavere ulcera plurima pure sania ac tabo manantia reperta sunt, variis partibus inusta, mesocolo intestinis omnibus crassioribus, sed unum colo extremo insederat, quod intestinum ipsum exederat et perforaverat, unde purulenta multa ex putrefactis prædicti mesocoli glandulis et vasis emanans et alvo inferiore coercita et cumulata trium librarum semisestariorum parisiensium mensuram implere poterat. Deprehensus quoque in rene dextro abcessus sed exiguus, et fermè nihil faciendus. In fundo ventriculi lientre abraso vicinis grandior et alii perexigui plures, et humoris fusci, fuliginosi atque ex viridi nigritantis copia insignis, quo, aut simili omnia ad unum intestina, usque ad extremum rectè referta erant.

"Vesicula fellea hepati subjecta et imis ejusdem partibus, affixa ab humore bilioso crassiore propè vacua. Hepar exsuccum planè ac retorridum....*simile* quod et duriusculi contra ventrem lanabat et solvebatur in grumos. Pulmonis sinistri lobus, pleuræ firmiori adherens et affixus ulcere maximo et profundissimo, pure plurimo confertus, et putrefactus apparuit."

Louis XIII. would thus appear to have died of phthisis pulmonalis with ulcerations of the bowels.

### Louis XIV.

This is a rather extraordinary dissection of an extraordinary being. At one time amongst the most fortunate of men, he was hailed as Louis le Grand, but before the close of his career he might justly have been termed Louis l'Infortuné. Born almost a fugitive, ruled by his mother Anne of Austria, who, in her turn, was beneath the thumb of the wily Mazarin; the education of Louis consisted almost exclusively in the study of the tragedies of Corneille. Hence that inordinate love of what the world calls glory,—hence that wanton waste of human blood for a favourite object, which rendered this prince in some degree a type of the reckless Napoleon,—hence all his greatness, and all his reverses. He obeyed, exiled, recalled, and again obeyed Cardinal Mazarin; made war through his general, the great Turenne; gained many battles, but risked his life in none; rooted out heresy by missionaries to convert, and dragoons to enthrall; fell successively under the influence of Madame de Montespan, and Madame de Maintenon; lived a superstitious man, and died a brave one. At the age of 74 he was operated on for fistula, and although the result was successful, he never regained his accustomed spirits. He now lost his only son, and within a few short months, the Duke of Burgundy, the Duchess his wife, and their eldest son, were all swept away and laid in the same tomb. Misfortunes abroad and miseries at home, a weakened empire and a discontented people dashed the last years of the old king with care, and at seventy-seven the vanity and ambition of his manhood were nearly quenched. At length the inevitable hour approached, and his spirit rose as his feeble frame sank. "Why do you weep," said he to one of his domestics, "did you think me immortal?" Bequeathing good counsel with his dying breath, the star of Louis XIV set for ever!

SECTIO CADAVERIS.—The whole of the left side of the body appeared gangrenous, from the extremity of the foot to the top of the head. The epidermis was generally detached from the cutis, the right side was gangrenous in several places, but less so than the left, and the belly was excessively blown up.

On opening the abdomen, the intestines, especially those on the left side, were found "altered," with some marks of inflammation; the large intestines were enormously dilated. In the left kidney was a small stone, similar to what the King had frequently voided during life, without any evidence of pain. The liver, spleen, stomach, and bladder, were healthy.

On opening the chest, the lungs were sound, as was the heart. The extremities of the blood-vessels, and some of the valves, were ossified; all the muscles of the throat were gangrenous.

On opening the head, the whole of the dura mater was found adherent to the cranium, and the pia mater had two or three *purulent spots* along the falx. The brain was otherwise healthy.

The left thigh internally was in a state of mortification, as were the muscles of the hypogastrium, and indeed this condition existed as high as the throat. The blood and lymph were *universally fluid* in the vessels.

The disease of which the King died, appears to have been an extreme degree of the gangrena senilis.

LOUIS XV.

After a reign of alternate benignity and cruelty, useful designs for the advancement of learning, and tyrannous measures to strangle freedom, conjugal fidelity in the commencement, and profligate debauchery in the sequel; Louis XV. fell a victim to his vices. He had been seduced from his Queen by the allurements of some ladies of his court; had been weaned from them by the avaricious and ambitious Madame de Pompadour; had abandoned her for the dissolute De Barré; and at length, when his jaded appetites ceased to be stimulated even by the meretricious arts of the latter, he resorted to mistress after mistress, and finally received his fatal malady, the small pox, from one who was infected with that disease. It was in this King's reign that the celebrated execution of Damien took place. No examination of the body of Louis was instituted, on the following singular, and somewhat ludicrous account.

The superstitious fears entertained regarding small pox completely drove the attendants in the palace from the body of the dead monarch. The first gentleman of the bedchamber, more faithful or more bold than the rest, demanded of Lamartinière, then chief surgeon, why he did not proceed to examine the corpse, and added, that he *must* do so. "My Lord Duke," answered Lamartinière, with his usual *brusquerie*, "your office renders it imperative upon you to hold the head of the deceased during the process. I declare to you, that if it is opened, neither you, nor I, nor any one of those assisting, will be alive eight days afterwards." Need we add, that Monsieur le Duc said no more about the matter!

The seeds of the revolution which had been sewn in the immoralities and arbitrary acts of the preceding reign, ripened into the unequalled horrors and atrocities of that of Louis XVI. With him the guillotine took the place of the scalpel, the executioner's report was substituted for that of the Dean of the Faculty of Medicine, the remains lay rotting in a lime pit, instead of reposing amidst the dust of Charlemagne and Henri Quatre, at St. Denis. Like the body of the Roman, it *vanished in the tempest!*

### Louis XVII.

The son of the last king never ascended the throne of France, but died whilst young, in the durance of the regicides and revolutionists. That no foul play, at least no overt violence, was inflicted, the account of the dissection, and the respectable names of Dumangin, Pelletan, Lassus, and Jeanroy are sufficient guaranty. The document signed by these gentlemen purports, that in pursuance to a warrant from the Committee of General Safety, they repaired to a second-floor apartment in a tower of the temple, where they found the body of the son of the deceased Louis Capet. He *appeared* to be about ten years old, and was known to two of the subscribed, who had attended him for *some days* during life. They were told that he had died at three o'clock on the preceding afternoon, and putrefaction was commencing on the belly, the scrotum, and inside of the thighs.

The whole frame bore the aspect of marasmus; the belly was tense and tympanitic. On the inside of the right knee was a tumour without change of colour of the skin, and another smaller tumour over the os radius near the left hand. The tumour of the knee contained about two ounces of greyish matter, a mixture of pus and lymph, situated between the periosteum and the muscles; that over the radius contained matter of the same kind, but more consistent.

On opening the abdomen, about a pint of yellowish and very fetid sero-purulent fluid flowed out. The intestines were blown up, pale, adherent to one another, and to the walls of the abdomen, and studded with a great number of tubercles of different sizes, which contained the same description of matter as that in the external tumours. The omentum and mesentery were filled with "lymphatic tubercles" like the former, and others were dispersed over the peritoneum. The interior of the stomach and intestines, the liver, the spleen, the pancreas, and the kidneys were sound.

The lungs adhered universally to the sides of the chest, the diaphragm, and the pericardium, but their substance was sound and only a few tubercles were found in the neighbourhood of the trachea and œsophagus. The heart and pericardium were natural. The brain and its appendages were also sound.

The reporters add that the disease of which this unfortunate young prince died was evidently chronic in its march and scrofulous in its nature. It was chronic inflammation and tubercular affection of the peritoneum. How far the tender mercies of the Conseil Général and the Sans Culottes may have contributed to light up this scrofulous affection is more than we shall presume to determine. God keep us and our's from the nursing of the Robespierres and Marats!

We now come to him who, like the rod of Aaron, swallowed up the memories of all his predecessors; who combined the great qualities of Charlemagne with the military despotism of Sylla, the deep selfishness of Marc Anthony, and a taste of the bloody vindictive cruelty of Marius; who had almost the daring of Alexander and more than the military skill of the first Cæsar; who fought his way to the throne of the world like a gallant man, ruled it like a great man, and lost it like a weak one; we come to Napoleon Buonaparte.

We have already detailed so fully the particulars of his demise and his subsequent examination, that no necessity remains for our noticing them again. We could not, however, pass by this extraordinary being in silence, or draw a veil before his niche in our pages, like that black daub that blots out the person of Marino Faliero from amongst the Doges of Venice in the Palace of Saint Mark.

### LOUIS XVIII.

No official account of the dissection of the last Louis has yet been published. The dean of the faculty, M. Landré-Beauvais, with those of the professors whose duty it is, were present on the occasion, and a notice of the particulars appeared in the Gazette de Santé, which has never been contradicted, and is therefore no doubt correct. The statement in question is thus prefaced :—"Although the procès-verbal has not been made public, we believe we may depend upon the accuracy of the following details."

SECTIO CADAVERIS. The bones of the anterior part of the skull were very thick, whilst those of the posterior part were thinner than usual. The brain was very large, but the left side was more developed than the right.

The lungs were perfectly healthy—the heart large, flabby, and empty of blood.

The stomach was very large, distended by gas and mucous matters, and it presented small red patches on its internal surface. The intestines were sound, but a steatomatous tumour of considerable size was found in the folds of the mesentery; it had occasioned no pain during life, and its existence had not been indicated by any perceptible symptoms. The other viscera were healthy.

The superior and inferior extremities were much wasted; the left thigh shewed on its inner side the mark of an ancient blister. Both legs, from the knees to the extremity of the feet, were converted into a yellow lardaceous substance, in which the cellular, the muscular, and even the osseous structures were confounded. A knife penetrated easily into the bones themselves. The right foot and the small of the leg were sphacelated, the bones softened, and four toes had been successively detached in the progress of the gangrene. The left foot was sphacelated likewise, but only as high as the tarsus.

From these details it is clear that Louis XVIII. like his greater ancestor the XIVth. of the name, was destroyed by gangrene of the lower extremities. Whether the gastronomic indulgences of the late monarch had any thing to do with the event, is scarcely problematical; but certain it is that the state of the lower extremities amply justifies the sarcasm of the Parisians on the restoration, who represented Louis as entering the capital in the form of a fat porker. Peace to his manes, for they sleep the quiet sleep—

> In that deep vault
> To whose foul mouth no healthsome air breathes in;
> Where for these many hundred years, the bones
> Of all his buried ancestors are packed :
> Where he, the last, as yet but green in earth,
> Lies fest'ring in his shroud.

We must now conclude this article, and we may add that the little pains we may have bestowed upon it will be amply rewarded if it affords either instruction or gratification to any of our readers.

---

